# The Reason

**Published:** 1870-04

**Authors:** 


### THE REASON.

Sometimes a newspaper bag falls into the river, or is burnt, or is missent, or from various other causes may never come to hand. Some time ago, a postmaster's cellar was found nearly full of newspaper mail-bags, the accumulations of months, from a wicked neglect of sworn official duty; one result of which was the writing of thousands of angry notes to publishers, for missing numbers, which loss they had to assume, without any thanks from their irate subscribers, who, no doubt, settled down in the belief that they were injured innocents, to the extent of an envelope, a sheet of paper, a three-cent stamp, and time and trouble of writing a letter, to say nothing of the discomfort of getting as mad as a March hare for not receiving their publication in due time.

Within a month of this same event, the entire paper mail from New York city was wholly destroyed by fire.

Sometimes publishers get out of papers, and it may not be convenient for a month or more to print a new edition; then don't get into a stew if you don't get your missing paper by return mail. Sometimes persons forget to sign their names, or to tell where they live, or fail to put a stamp on their letters.

Moral. — In the light of these facts, when you fail to receive your paper once, or twice, or oftener, don't make a fool of yourself by writing an angry note, but simply notify your publisher that you have not received your paper, and if you give your address in full, town, county, and State, and write your own name, without any flourishes or quirly-me-jigs, you will pretty certainly receive your missing number in a reasonable time.

